# Repression of telomere-associated genes by microglia activation in neuropsychiatric disease

**DOI:** 10.1007/s00406-016-0750-1

**Published:** 2016-11-28

**Authors:** Golo Kronenberg, Ria Uhlemann, Johanna Schöner, Stephanie Wegner, Valérie Boujon, Nikolas Deigendesch, Matthias Endres, Karen Gertz

**Affiliations:** 1 Klinik für Psychiatrie und Psychotherapie, Charité Campus Mitte, Berlin, Germany; 2 Center for Stroke Research Berlin (CSB), Charité - Universitätsmedizin, Berlin, Germany; 3 Klinik und Hochschulambulanz für Neurologie, Charité - Universitätsmedizin, Berlin, Germany; 4 Klinik und Poliklinik für Psychiatrie und Psychotherapie, Universitätsmedizin Rostock, Gehlsheimer Straße 20, 18147 Rostock, Germany; 5 Institut für Neuropathologie, Charité - Universitätsmedizin, Berlin, Germany; 6 German Center for Neurodegenerative Diseases (DZNE), Charitéplatz 1, 10117 Berlin, Germany; 7 Cluster of Excellence NeuroCure, Charité - Universitätsmedizin, Berlin, Germany; 8 DZHK (German Centre for Cardiovascular Research), Partner Site Berlin, Charitéplatz 1, 10117 Berlin, Germany

**Keywords:** Alzheimer’s disease, Microglia, Mitochondrial biogenesis, Neurodegenerative disease, Telomerase

## Abstract

Microglia senescence may promote neuropsychiatric disease. This prompted us to examine the relationship between microglia activation states and telomere biology. A panel of candidate genes associated with telomere maintenance, mitochondrial biogenesis, and cell-cycle regulation were investigated in M1- and M2-polarized microglia in vitro as well as in MACS-purified CD11b+ microglia/brain macrophages from models of stroke, Alzheimer’s disease, and chronic stress. M1 polarization, ischemia, and Alzheimer pathology elicited a strikingly similar transcriptomic profile with, in particular, reduced expression of murine *Tert*. Our results link classical microglia activation with repression of telomere-associated genes, suggesting a new mechanism underlying microglia dysfunction.

## Introduction

Telomere dysfunction has been implicated in cellular senescence and pathological aging. Apart from its canonical role in telomere extension in dividing cells, TERT, the catalytic subunit of telomerase, has also been shown to interact with mitochondrial proteins [1]. Mice null for telomerase reverse transcriptase display repression of peroxisome proliferator-activated receptor ɣ coactivator 1 α and β (*PGC*-*1α* and *PGC*-*1β*), metabolic compromise and reduced mitochondrial biogenesis and function (“PGC network”; [2]).

Microglia/brain macrophages play a crucial role in neurodegenerative and neuropsychiatric disease. Traditionally, two main patterns of microglia activation are distinguished: the so-called M1 (classical, LPS-induced) and M2 phenotypes (“alternatively activated,” stimulated by IL-4). Here, we studied the PGC gene network in M1 and M2 microglia in vitro as well as in ex vivo MACS-purified CD11b+ microglia/macrophages from three disease models, namely: transient brain ischemia [3], Alzheimer’s-like pathology [4], and a chronic stress paradigm used to elicit anxious/depressive-like behaviors [5–7].

## Materials and methods

### Animals and treatments

All experimental procedures were approved by the respective official committees and carried out in strict accordance with the Animal Welfare Act, the European Communities Council Directive of November 24, 1986 (86/609/EEC) and the ARRIVE (Animals in Research: Reporting In Vivo Experiments) guidelines [8]. Male 129/SV mice were 7–8 weeks old and weighed between 18 and 22 g at the beginning of experiments. APPPS1 mice [4] and wild-type littermate controls were 6 months old at the time of the experiments. Animals were housed in standard mouse cages in groups of 4–6 mice per cage at 22–23 °C with a standard light–dark cycle (7 AM–7 PM). Animals were randomized to experimental groups. Transient brain ischemia was induced by 30 min left filamentous middle cerebral artery occlusion (MCAo)/reperfusion as reported earlier [9]. The chronic stress procedure spanned 28 days and was carried out as described at length previously [6]. Briefly, the procedure consists of exposure to a rat, restraint stress, and tail suspension, which were applied in the following sequence: days 1–7, exposure to a rat; days 8–10, restraint stress; days 11–14, tail suspension; days 15–21, exposure to a rat; days 22–25, restraint stress; and days 26–28, tail suspension.

### Microglia cultures

Cultures of primary murine microglia were prepared from newborn C57Bl6 mice (P0–3) as described previously [10, 11]. In brief, microglial cells were harvested by gentle shake-off and seeded at an initial density of 10^6^ cells/ml. Cells remained in culture for additional 24 h before use. The purity of cultures exceeded 98%, which was confirmed by regular flow cytometry analyses with CD11b and CD45 staining (rat anti-mouse CD11b #553312 and rat anti-mouse CD45 #553081: both from BD Biosciences). All experiments were performed in DMEM containing 10% fetal calf serum, 1% Pen/Strep, 1% sodium-pyruvate and 4.5 g/l d-glucose (“complete medium”; all from Biochrom/Merck KGaA). Recombinant murine IL-4 (PeproTech) was used at a concentration of 10 ng/ml [10]. LPS (Escherichia coli 055:B5, Sigma-Aldrich) was applied at a concentration of 1 μg/ml [11].

### Ex vivo isolation of adult mouse microglia

All kits were from Miltenyi Biotec. Adult mice were perfused transcardially with 0.9% saline. After quick removal, brains were dissociated using the Neural Tissue Dissociation Kit (P) according to the manufacturer’s instructions. After dissociation, myelin was eliminated using Myelin Removal Beads. Finally, for magnetic cell sorting (“MACS”) via columns, the cell suspension was incubated with CD11b MicroBeads. In the stroke experiments, microglia/macrophages were harvested from the infarcted tissue of the ipsilateral hemisphere (MCA territory). In the other experiments, whole brains including cerebellum were used.

### Messenger RNA isolation and quantitative polymerase chain reactions

We followed established protocols for mRNA isolation and quantitative polymerase chain reactions [3]. Total RNA was extracted using the NucleoSpin^®^ Tissue XS kit (Macherey–Nagel). For PCR amplification, we used gene-specific primers (Table 1) and Light Cycler^®^ 480 SYBR Green I Master (Roche Diagnostics). Polymerase chain reaction conditions were as follows: preincubation 95 °C, 10 min; 95 °C, 10 s, primer-specific annealing temperature, 10 s, 72 °C, 15 s (45 cycles). Crossing points of amplified products were determined using the Second Derivative Maximum Method (Light Cycler 480 Version 1.5.0, Roche). Quantification of messenger RNA expression was relative to tripeptidyl peptidase (Tpp) 2 [12]. The specificity of polymerase chain reaction products was checked using melting curve analysis.

**Table 1:** Primer sequences used in quantitative real-time polymerase chain reactions

Primer	For	Rev
*Nrf1*	cca cgt tac agg gcg gtg aa	agt ggc tcc ctg ctg cat ct
*Nfe2I2* = *Nrf2*	gca cag aag aaa gca ttg tg	agt gtg gtg agg tct ata tc
*PGC1α*	cac gca gcc cta ttc att gtt cg	gct tct cgt gct ctt tgc ggt at
*PGC1β*	caa cta tct ctc tga cac gca g	ctc act gtc aat ctg gaa gag c
*Tfam*	ctt cga ttt tcc aca gaa cag c	ctt tgt atg ctt tcc act cag c
*Terf1*	ctt tcg tcg tac tcg tga cag	gag ttc caa atc atc agg gct g
*Terf2*	cac acc ctt gga atc agc tat c	gtt cag gag atc agt tct cag c
*Tert*	gtt gcc caa tgc cta gtg tgc	cac tcg gct caa cag tag cat c
*Chek2*	caa gaa cct gaa gaa cct ggt c	gct cgg tat tta cga agg ttc c
*Trp53*	gac agc caa gtc tgt tat gtg c	gtc ttc cag ata ctc ggg ata c
*Cdkn1a* = *P21*	gtg gaa ctt tga ctt cgt cac g	caa tct gcg ctt gga gtg ata g

### Statistical analysis

**Fig. 1:**
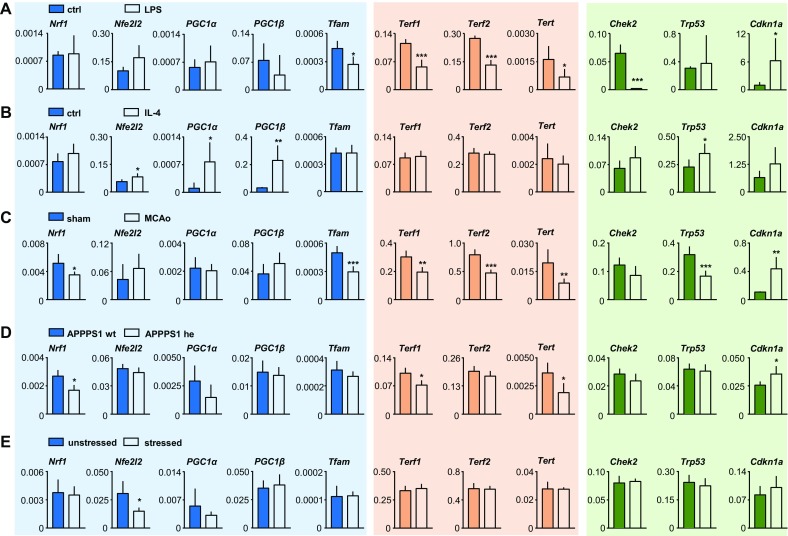
**a** Primary postnatal microglia cultures were treated with LPS (1 µg/ml, 6 h). *N* = 4–5 independent measurements per data point. **b** Primary postnatal microglia cultures were treated with IL-4 (10 ng/ml, 24 h). *N* = 4–5 independent measurements per data point. **c**. After 7 days, CD11b+ cells were MACS-sorted from the brains of mice subjected to 30 min MCAo/reperfusion or to sham operation. *N* = 5–6 animals per group. **d** CD11b+ cells were MACS-sorted from the brain of 6-month-old APPPS1 animals and compared to wild-type littermates. *N* = 4 mice per group. **e** Upon completion of the chronic stress procedure, mice were sacrificed and CD11b+ cells were MACS-sorted from the brain. *N* = 5–6 mice per group. *p < 0.05, **p < 0.001, ***p < 0.0001

Experiments were carried out in a blinded fashion. Data are presented as mean ± SD. Groups were compared by unpaired *t* test with level of significance set at 0.05 and two-tailed p values using Graph-Pad Prism 6 (GraphdPad Software). For data sets which were not normally distributed, nonparametric testing was performed using the Mann–Whitney test (*Cdkn1a* in Fig. 1a, *PGC1α* in Fig. 1b, *Terf1* in Fig. 1c). Analyses of LPS- and IL-4 stimulated primary murine microglia are based on 4–5 independent samples per condition (Fig. 1a, b). Analyses of CD11b+ adult microglia/brain macrophages are based on five sham-operated and six MCAo mice (stroke condition; Fig. 1c), 4 APPPS1 mice and 4 littermate controls (Alzheimer’s model; Fig. 1d). In the stress condition, a sample of Cd11b+ MACS-sorted cells was pooled from 2 to 3 mice with 5 independent samples for the stress group and 6 independent samples for the unstressed control group (Fig. 1e).

## Results

A panel of key marker genes associated with mitochondrial biogenesis (*Nrf1*, *Nfe2l2*, *PGC1α*, *PGC1β*, *Tfam*), the telomere complex (*Terf1*, *Terf2*, *Tert*) and cell-cycle regulation (*Chek2*, *Trp53*, *Cdkn1a*) were investigated.

First, we studied gene expression in cultured primary murine microglia (Fig. 1a, b). LPS stimulation (Fig. 1a) and IL-4 stimulation (Fig. 1b) represent the two extremes of microglia polarization in vitro. Interestingly, the patterns of gene regulation differed profoundly between either condition. M1 microglia showed robust downregulation of *Tfam* as well as of genes associated with the telomere complex (*Terf1*, *Terf2*, *Tert*). Moreover, mRNA expression of two of the cell-cycle regulators was strongly affected by LPS stimulation (Fig. 1a). M2-polarized, alternatively activated microglia showed an entirely different pattern with upregulation of several genes associated with mitochondrial biogenesis and energy metabolism (*Nfe2l2*, *PGC1α*, *PGC1β*) as well as upregulation of tumor suppressor *Trp53* (Fig. 1b).

Next, we studied gene expression in ex vivo isolated CD11b+ adult microglia/brain macrophages. The following disease conditions were investigated: transient mild brain ischemia (Fig. 1c), a murine model of Alzheimer’s disease (Fig. 1d) as well as a 4-week chronic stress paradigm (Fig. 1e). Strikingly, the pattern of effects observed in the ischemic brain 7 days after 30 min MCAo/reperfusion closely recapitulated the findings in LPS-stimulated microglia in vitro with downregulation of *Tfam*, *Terf1*, *Terf2*, *Tert*, and upregulation of *Cdkn1a* (Fig. 1c). A similar, albeit weaker, pattern of effects also emerged in Alzheimer’s-like brain with significant downregulation of *Terf1*, *Tert*, and upregulation of *Cdkn1a* (Fig. 1d). The 4-week stress paradigm did not exert strong effects on any of the telomere-associated molecules. Similarly, there was no apparent effect of chronic stress on cell-cycle regulation. *Nfe2l2* mRNA expression was decreased in ex vivo isolated CD11b+ cells following chronic stress.

## Discussion

Along with monocytes invading the brain parenchyma after injury, microglia constitute the main cellular effectors of innate immunity in the central nervous system. Activated microglia fulfill a plethora of functions including detection and removal of pathogens and debris, antigen presentation, secretion of cytokines and chemokines, resolution of neuroinflammation, and modulation of brain repair, e.g., by releasing neurotrophic factors [13]. In a relatively recent and surprising paradigm shift, microglia have come to the fore as key players across a wide range of neurological and neuropsychiatric disorders, in particular disorders related to aging such as stroke or Alzheimer’s disease [14, 15].

In the current study, we examined the effects of M1 and M2 polarization of cultured murine microglia on a panel of key marker genes associated with the telomere complex, mitochondrial function, and cell-cycle regulation (PGC network; [2]). Then, we compared these ideal-typical patterns with mRNA regulation in MACS-sorted microglia/macrophages harvested from the brain of adult mice subjected to mild transient ischemia, chronic stress, or expressing mutant amyloid precursor protein. It should be specifically noted that all in vivo models investigated here represent subacute or chronic changes in activation states (i.e., 7 days after middle cerebral artery occlusion/reperfusion; 6-month-old APPPS1 mice with established amyloid plaques [4]; 4-week stress model). We anticipate that the mRNA results presented here will serve as a reference for future studies of the PGC network in microglia/brain macrophages under different physiological and disease conditions.

The strongest effects on our panel of candidate genes were observed after LPS stimulation of microglia in vitro, supporting the notion that along with a pronounced metabolic shift (e.g., [10, 11, 16]), classical microglia activation elicits a robust transcriptomic response. The effects of IL-4 followed an entirely different pattern from that of LPS. Furthermore, generally speaking, the effects of chronic stress were modest. By contrast, brain ischemia and, to a lesser degree, Alzheimer’s-like pathology yielded relatively similar patterns of mRNA changes to those observed after stimulation with LPS.

The most striking and unexpected finding of this study is that M1 polarization strongly represses genes associated with the telomere complex. Importantly, both ischemia and Alzheimer’s-like pathology recapitulated this cell type-specific pattern of reduced Tert mRNA expression in vivo. A good correlation between telomerase activity and Tert mRNA expression has previously been reported (e.g., [17, 18]). It is therefore likely that the transcriptomic changes observed here contribute directly to microglial cellular dystrophy and senescence such as is observed during aging and in aging-related neurodegenerative diseases (e.g., [19, 20]).
